# Efficient and reproducible depletion of hepatitis B virus from plasma derived extracellular vesicles

**DOI:** 10.1002/jev2.12040

**Published:** 2020-12-22

**Authors:** Stephanie Jung, Karolin Fiona Kirsten Jacobs, Mikhail Shein, Anne Kathrin Schütz, Fabian Mohr, Herbert Stadler, Daniela Stadler, Aaron Michael Lucko, Sebastian Maximilian Altstetter, Florian Wilsch, Li Deng, Ulrike Protzer

**Affiliations:** ^1^ Institute of Virology School of Medicine Helmholtz Zentrum München/Technical University of Munich Garching Germany; ^2^ Bavarian NMR Center, Department of Chemistry Technical University of Munich Garching Germany; ^3^ IBA GmbH Göttingen Germany; ^4^ German Center for Infection Research (DZIF) Munich partner site Garching Germany

**Keywords:** affinity‐based purification, EV purification, extracellular vesicles, hepatitis B virus, size‐exclusion chromatography, virus removal

## Abstract

Extracellular vesicles (EVs) are emerging fundamental players in viral infections by shuttling viral components, mediating immune responses and likely the spread of the virus. However, the obstacles involved in purifying EVs and removing contaminating viral particles in a reliable and effective manner bottlenecks the full potential for the development of clinical and diagnostic treatment options targeting EV. Because of the similarities in size, density, membrane composition and mode of biogenesis of EVs and virions there are no standardized approaches for virus‐removal from EV preparations yet. Functional EV studies also require EV samples that are devoid of antibody contaminants. Consequently, the study of EVs in virology needs reliable and effective protocols to purify EVs and remove contaminating antibodies and viral particles. Here, we established a protocol for EV purification from hepatitis B virus (HBV)‐containing plasma by a combination of size‐exclusion chromatography and affinity‐based purification. After purification, EV samples were free of virus‐sized particles, HBV surface antigen, HBV core antigen, antibodies or infectious material. Viral genomic contamination was also decreased following purification. By using appropriate antibodies and size parameters, this protocol could potentially be applied to purification of EVs from other viral samples. In summary, we established a fast, reproducible and robust approach for the removal of HBV from EV preparations. Looking forward to the point of purifying EVs from clinical samples, this method should enable studies shedding light on the underlying mechanisms of EVs in viral infections and their diagnostic and prognostic potential.

## 1 INTRODUCTION

Extracellular vesicles (EVs) is a collective term for membranous particles actively released by living cells. This heterogeneous group comprises diverse members varying in the mode of biogenesis and size, including multivesicular‐body derived exosomes (40–100 nm), microvesicles budding on the plasma membrane (50 nm–1 μm), and larger apoptotic bodies (van Niel al., 2018). EVs enable intercellular communication by selective shuttling of signalling molecules like nucleic acids, proteins and cytokines between donor and recipient cells (Kouwaki et al., 2017). Many publications report the EV‐mediated transfer of viral components. This transfer results in pro‐ and antiviral effects by both activating and inhibiting immunity to infections (Bukong et al., 2014; Cai et al., 2018; Crenshaw et al., 2018; Dreux et al., 2012; Jung et al., 2020; Li et al., 2013; Longatti; 2015; Ramakrishnaiah et al., 2013; Sampey et al., 2016; Schwab et al., 2015, Yang et al., 2017). EVs have even been proposed to encapsulate whole viruses and viral genomes (Trojan exosome) and thereby would contribute to shielding viral particles from antibodies and assist with viral spread (Altan‐Bonnet, 2016; Feng et al., 2013, 2015; Morris‐Love et al., 2019; Raab‐Traub & Dittmer, 2017). EV‐mediated transfer of infectious viruses could potentially circumvent antibody responses, worsening clinical outcomes and diminishing the efficacy of vaccinations. Further research to identify the impact of EVs on viral infections is required, but is severely impaired by the unavailability of virus‐EV separation methods (Urbanelli et al., 2019).

Hepatitis B virus (HBV) infections is a global health threat, affecting more than 250 million chronical carriers and accounting for nearly 900,000 deaths/year (World Health Organization, 2017). Although there is a great need for successful treatment options, no therapy leading to viral clearance has been approved for clinical use so far (Ko et al., 2017). This situation is further complicated by the fact that the interaction of HBV with the immune system is not sufficiently understood (Jung et al., 2020). Further research in this area is urgently required, and the question arises how EVs contribute to HBV pathogenesis.

The challenge of purifying virus‐free EVs from plasma is largely due to the overlaps of EVs and viruses in size, density and enveloping membranes (Nolte‐’t Hoen et al., 2016; Raab‐Traub & Dittmer, 2017), as well as the heterogenous composition of plasma samples including various plasma proteins (Karimi et al., 2018; Simonsen, 2017). The usage of specific surface markers and release inhibitors is further complicated as both viruses and EVs bud on the plasma membrane, or into multivesicular bodies, using similar release mechanisms, which severely limits the spectrum of potential experiments. Therefore, the purification of virus‐free EVs is necessary for the effective study of EV‐mediated viral protection and mobility.

Previously published approaches on virus removal from EV samples include NanoFACS and affinity‐based positive selection, but none have rendered pure EVs without bound antibodies (Barclay et al., 2019; McNamara & Dittmer, 2019). Negative selection using a flavivirus‐group antigen targeting antibody 4G2 was not able to completely eliminate infectious potential from Dengue virus infected plasma (Martins et al., 2018). Furthermore, the absence of residual antibody or extensive controls for viral contaminations were not addressed in the study (Martins et al., 2018). Reiter and colleagues reported successful enrichment of EVs or virus‐like particles in separate chromatographic fractions (Burwitz et al., 2017). However, the authors did not claim a complete removal of virions from EV samples (Reiter et al., 2019).

Here, we developed a method for efficient removal of HBV from EV samples. The concentration of HBV in EV‐containing plasma samples were reduced firstly using size‐exclusion chromatography, by exploiting their small diameter (42 nm) (Liu, 2014). Secondly, a further complete removal of HBV was achieved using affinity‐based purification. Purity of the EV samples was verified through size measurement, EV‐specific protein staining in Western blot, and electron microscopy according to the recommendations of the International Society for Extracellular Vesicles (Thery et al., 2018).

## 2 MATERIALS AND METHODS

### 2.1 Sample sources

Blood samples were drawn from healthy human donors after informed consent and approval by the local ethics committee.

### 2.2 Antibodies and reagents

Western blot samples were lysed in Pierce RIPA Buffer (Thermo Scientific, Waltham, USA). For protein measurement, BCA Protein Assay (Thermo Scientific, Waltham, USA) was used.

Primary antibodies targeting Syntenin (clone EPR8102, 1:1000 diluted) were purchased from Abcam (Cambridge, UK), anti‐CD63 antibodies (cat. no. EXOAB‐CD63A‐1, 1:2500 diluted) were from SBI (Palo Alto, California, USA), anti‐TSG101 (cat. no. 5701, 1:1000 diluted) from Sigma (Taufkirchen, Germany). Non‐EV proteins were stained using anti‐Calnexin (cat. no. 610523, 1:1000 diluted, BD Biosciences, San Jose, California, USA) and anti‐albumin antibodies (cat. no. Ab8940, 1:1000 diluted, Abcam, Berlin, Germany). Secondary antibodies (anti‐mouse IgG Peroxidase, cat. no. A0168 and anti‐rabbit IgG Peroxidase, cat. no. A0545) were purchased from Sigma‐Aldrich (St. Louis, Missouri, USA) and diluted 1:10,000. Hepatitis B core Antigen targeting antibody (anti‐HBcAg; cat. no. B0586, 1:1000 diluted) was purchased from Dako (Hamburg, Germany). Hepatitis B virus Surface Ad/Ay Polyclonal Antibody (cat. no. PA1‐73080, 1:10 diluted) was used for affinity‐based purification and purchased from Invitrogen (Carlsbad, California, USA). Recombinant HBV was produced as previously described and purified via heparin binding columns and cesium chloride gradient centrifugation (Burwitz et al., 2017).

### 2.3 Size‐exclusion chromatography (SEC)

Plasma samples were prepared for EV purification as previously described (Lacroix et al., 2013) using citrated plastic tubes for blood drawing. Before SEC, 180 μl of plasma was diluted to 300 μl with phosphate‐buffered saline (PBS) or HBV stocks and hepatitis B surface antigen targeting antibody (anti‐HBsAg) as specified in the results section. Samples were incubated for 30 min at room temperature. EV purification was performed using qEVoriginal columns (Izon Science, Oxford, UK) with the following modifications to the manufactures instructions: (A) Sample input volume was decreased to 300 μl; (B) The column was rinsed with 2.7 ml PBS (total void volume 3 ml); (C) Immediately after elution of the void volume, fraction 1 (F1) was eluted in 1 ml PBS and fraction 2 (F2) was eluted in 500 μl PBS. EV fractions were subjected to immediate further applications or stored at −80°C.

### 2.4 Affinity‐based purification

3 ml Empty Straight‐Barrel Tubes (Biocomma, Shenzhen, China) were filled with PBS and enclosed by a lower frit that was previously de‐aired in 80% ethanol. Columns were loaded with 500 μl Protein A Agarose 50% suspension (IBA, Göttingen, Germany) and subsequently emptied by gravity flow. Samples with HBV already bound by polyclonal anti‐HBsAg were loaded into the column which was closed by upper and lower cap. Binding of anti‐HBsAg by protein A occurred within 6 min at room temperature under gentle inversion of the columns. Samples were retrieved by gravity flow and subjected to immediate further applications or stored at −80°C.

### 2.5 Dynamic light scattering

Particle size‐distribution measurement was performed by dynamic light scattering (DLS) using a Zetasizer^TM^ Nano ZS machine (ZEN3600, Malvern, Kassel, Germany) with detection at an angle of 173° (back‐scattering). Samples were diluted in 10 mM NaCl with a dilution factor of 10 and measured in disposable cuvettes (Malvern). Samples were run consecutively in triplicates for 50 × 10 s at 20°C. Instrument was set to automatic attenuation and positioning. A refractive index of 1.39 and sample absorption of 0.01 were assumed for all EV‐measurements. High measurement quality was assured by analysing raw correlation data, the resulting correlation fits, and polydispersity indices.

### 2.6 Nanotracking analysis

Samples were diluted at 1:10 in 0.02 μm filter‐sterilized PBS and particle concentration was determined using a NanoSight™ NS300 instrument (Malvern, Worchestershire, UK). Software version number NTA 3.3 was used for data acquisition and processing. Capture settings were 25 frames per second, with 749 frames total. Camera type was sCMOS with the following settings: (1) Laser Type Blue488; (2) Camera Level 14. Video analysis settings were set to a detection threshold of 5.

### 2.7 Negative stain transmission electron microscopy

Staining protocol: A 3 mm copper grid (300 mesh) with continuous carbon film support was glow discharged for 30 s at a current of 5 mA. 5 μL of the exosome sample was pipetted onto the grid and allowed to adsorb for 5 min. Residual solution was removed by filter paper (Whatman, grade 1) and the sample was stained with 2% uranyl acetate solution for 30 s. After removal of the staining solution, the grid was left to dry prior to measurements. Transmission electron microscopy (TEM): Micrographs were collected with a 60,000 fold magnification (0.275 nm/pix) using a JEOL JEM‐1400 Plus with a JEOL CCD Ruby camera (8 Mpix). The underfocus was set to 500 nm and voltage to 120 kV.

### 2.8 HBV infection

Expression of Na^+^‐taurocholate cotransporting popypeptide (NTCP) as an essential receptor for HBV uptake renders hepatoma cell lines susceptible for HBV infection (Yan et al., 2012). NTCP‐expressing HepG2‐cells (HepG2‐NTCP) were infected with purified EV samples or HBV as described previously (Ko et al., 2018). Infection rates 5 days post‐infection were determined by Hepatitis B early antigen (HBeAg) measurement in enzyme‐linked immunosorbent assay (ELISA) using Enzymegnost^®^ HBe monoclonal (Siemens Healthcare, Marburg, Germany).

### 2.9 Architect measurement

Hepatitis B surface antigen (HBsAg) was measured by Architect (Abbott Laboratories, Illinois, USA) using Architect HBsAg 6C36. The detection antibody is produced in goats (polyclonal) and shows activity for HBsAg subtype ad/ay.

### 2.10 HBV qPCR

HBV DNA was extracted from purified samples using NucleoSpin^®^ Tissue kit (Macherey Nagel, Düren, Germany) applying the support protocol for genomic DNA and viral DNA from blood samples. Total HBV DNA (primers: HBV 1745: GGAGGGATACATAGAGGTTCCTTGA, HBV 1844: GTTGCCCGTTTGTCCTCTAATTC) was determined by qPCR on a LightCycler 480 Real‐time PCR system and data was analysed via the LightCycler 480 Software 1.5.1.62 by absolute quantification using an HBV plasmid standard. Per reaction, 4 μl extracted DNA were mixed with 0.5 μl forward primer (20 μM), 0.5 μl reverse primer (20 μM) and 5 μl LightCycler 480 SYBR Green I Master mix. Conditions for the qPCR were: (A) initial denaturation at 95°C for 5 min. (B) 40 cycles of denaturation at 95°C for 25 s, annealing at 60°C for 10 s and elongation at 72°C for 30 s.

### 2.11 Statistical analysis

If not stated otherwise, data are presented as arithmetic means and SD. Statistical analyses of the generally normally distributed data were based on paired t‐tests. D'Agostino‐Pearson test was used as a test of normal distribution.

### 2.12 Deposited data

We have submitted all relevant data of our experiments to the EV‐TRACK knowledgebase (EV‐TRACK ID: EV200040) (Van Deun et al., 2017).

## 3 RESULTS

### 3.1 General procedure

**FIGURE 1:**
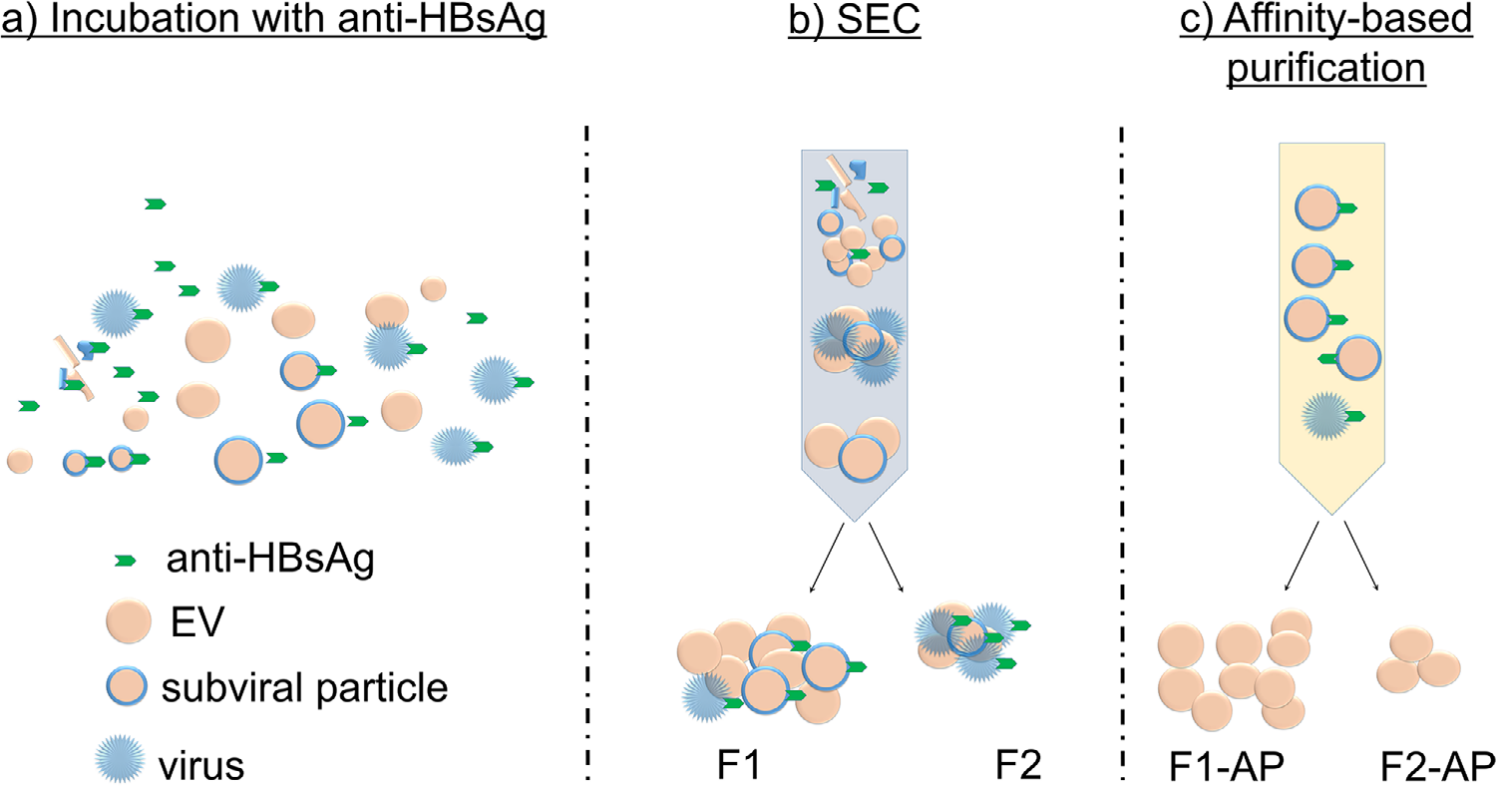
Schematic representation of virus removal from extracellular vesicle samples. (a) HBV containing plasma samples were incubated with anti‐HBsAg. (b) Samples were purified accordingly by SEC, leading to the removal of both unbound antibody and a decrease in HBV concentration (fractions F1 and F2). (c) HBV virions and subviral particles were removed in affinity‐based purification, and EV samples F1‐AP and F2‐AP were collected

We aimed to establish a method for the removal of virions from EV samples. Firstly, plasma samples containing EVs, HBV‐virions and subviral particles were incubated with anti‐HBsAg (Figure 1a). EVs were purified by SEC to remove unbound anti‐HBsAg, serum impurities, and the majority of HBV virions (Figure 1b). Complete depletion of anti‐HBsAg bound particles, such as virions and filaments, from EV preparations was obtained by affinity‐based purification (Figure 1c).

### 3.2 Removal of HBV from plasma samples by SEC

**FIGURE 2:**
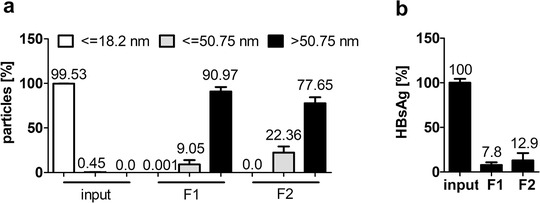
Hepatitis B virus was removed from EV‐containing plasma samples by size‐exclusion chromatography. Plasma samples containing 3.1 × 10^8^ GE HBV per ml plasma were subjected to SEC. (a) Size‐distribution of input material and two fractions of SEC‐purified plasma samples (F1 and F2) determined by Zetasizer measurement. Bars represent particles detected in the respective fraction in percentage of total sample according to size determined in three independent experiments in triplicates each. (b) HBsAg determined in input (plasma + HBV) and SEC‐purified plasma samples is given as a percentage of input concentration. Samples correspond to 60 μl starting plasma each. Data represent three independent experiments in duplicates

Particle size distribution measurement showed that the plasma‐derived input sample primarily consisted of antibody‐sized particles (≤18.2 nm) and contained more virus‐sized particles (≤50.75 nm) than EV‐sized particles (> 50.75 nm) (Figure 2a and Figure S2 and 6). As described in the methods part, plasma samples containing 3.1 × 10^8^ genome equivalents (GE) HBV per ml plasma (equals 5.56 × 10^7^ GE HBV and 180 μl plasma per 300 μl input sample) were subjected to SEC. Fractions F1 and F2 were greatly enriched in EV‐sized particles and virus‐sized particles were comparatively reduced. Additionally, SEC profoundly decreased HBsAg concentrations in a correlating manner (Figure 2b), after accounting for purification‐based dilution factors (Figure S1). However, it did not completely remove viral components.

### 3.3 Capacity of affinity‐based purification to remove HBV contaminations from EV samples

Affinity‐based purification was used as a supplementary step to remove residual HBV from EVs following SEC. In the first instance, antibody binding capacity of the affinity column was determined. In order to generate input samples for this titration experiment, EVs were purified by SEC according to the protocol described in the methods part and F1 was collected for downstream applications. The initial SEC allowed a clear differentiation between EVs and virions based on their size, since other plasma components in the size range of viral particles, such as very low density lipoproteins (Karimi et al., 2018; Simonsen, 2017), were no longer contained in the input sample.

Purified EVs were mixed with 1.54 × 10^7^ GE HBV and 75.5 μg anti‐HBsAg per ml input sample and affinity columns were incubated with input sample to determine their antibody‐binding capacity. Input fractions were gradually added by 1 ml at a time, so increasing amounts of total antibody load were bound to the column and flow through of fractions was collected separately.

**FIGURE 3:**
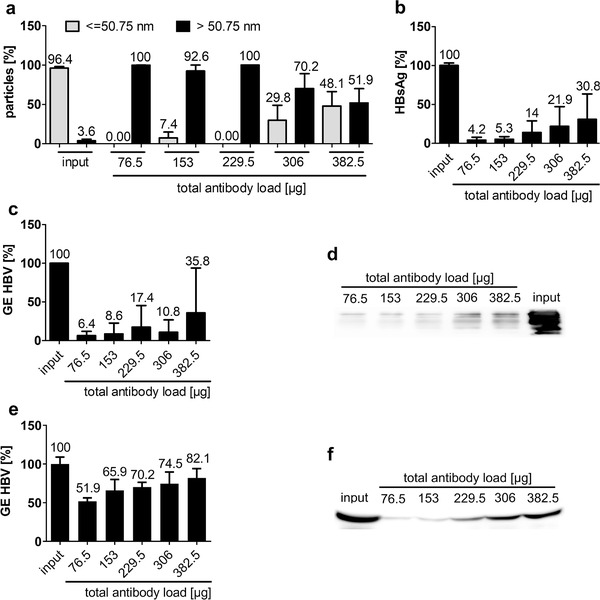
Hepatitis B virus was removed from EV samples by affinity‐based purification. (a‐d) Input samples containing EV purified from 67 μl plasma, 1.54 × 10^7^ GE HBV and 76.5 μg anti‐HBsAg per ml input sample were subjected to affinity‐based purification. Increasing amounts of anti‐HBsAg were gradually bound to the column as indicated in the figure. (a) Size‐distribution of input material and affinity‐purified fractions determined by ZetasizerM measurement. Bars represent particles detected in the respective fraction in percentage of total sample according to size determined in three independent experiments in triplicates each. (b) HBsAg determined in input (EVs, HBV and anti‐HBsAg) and affinity‐purified fractions is given as a percentage of input concentration. Samples correspond to 6.7 μl starting plasma each. Data represent three independent experiments in duplicates. (c) GE HBV determined in input and affinity‐purified fractions by qRT‐PCR is given as a percentage of input concentration. Samples correspond to 13.4 μl starting plasma each. Data represent three independent experiments in technical triplicates. (d) Western blot analysis of residual anti‐HBsAg contaminations. Samples correspond to 1.34 μl starting plasma each. One representative Western blot out of three independent experiments is shown. (e,f) Input samples containing 3.1 × 10^8^ GE HBV and 767 μg anti‐HBsAg per ml plasma were subjected to affinity‐based purification. Increasing amounts of anti‐HBsAg were gradually bound to the column as indicated in the figure. (e) GE HBV determined in input and affinity‐purified fractions by qRT‐PCR is given as a percentage of input concentration. Samples correspond to 13.4 μl starting plasma each. Data represent three independent experiments in technical triplicates. (f) Western blot analysis of residual anti‐HBsAg contaminations. Samples correspond to 1.34 μl starting plasma each. One representative Western Blot out of three independent experiments is shown

Percentage of HBV‐sized particles, HBsAg and HBV GE were greatly reduced in early fractions (fraction 1: 76.5 μg total antibody load, EVs purified from 67 μl plasma, 1.54 × 10^7^ GE HBV; fraction 2: 153 μg total antibody load, EVs purified from 134 μl plasma, 3.1 × 10^7^ GE HBV) but increased in later fractions after the column became overloaded (Figure 3a‐c and Figure S3). Elution of unbound antibodies was low in early fractions, but increased in later fractions (Figure 3d). Consequently, 153 μg of anti‐HBsAg were subsequently considered the maximum column binding capacity. In order to ensure comparability to further purification steps, affinity‐based purification was repeated with plasma samples containing 3.1 × 10^8^ GE HBV and 767 μg anti‐HBsAg per ml starting plasma. Removal of HBV GE (Figure 3e) and anti‐HBsAg (Figure 3f) was detectable but less efficient than removal of these components from pre‐purified samples (compare Figure 3c and d). Therefore, we consider pre‐purification prior to affinity‐based purification as essential.

### 3.4 Combination of SEC and affinity‐based purification optimizes HBV removal from plasma samples

**FIGURE 4:**
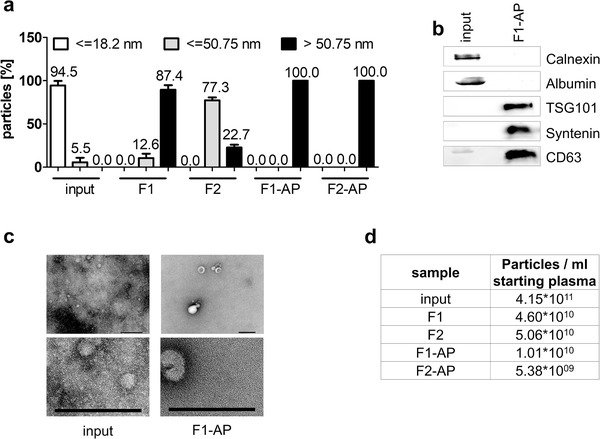
Combination of size‐exclusion chromatography and affinity‐based purification results in pure EV samples. Plasma samples containing 3.1 × 10^8^ GE HBV and 767 μg anti‐HBsAg per ml plasma were subjected to SEC and affinity‐based purification. (a) Size‐distribution of input material and purified fractions determined by Zetasizer measurement. Bars represent particles detected in the respective fraction in percentage of total sample according to size determined in three independent experiments in triplicates each. (b) Western blot analysis of calnexin and albumin (non‐EV proteins) and tumour susceptibility gene 101 (TSG101), cluster of differentiation (CD) 63 and syntenin (EV‐enriched proteins). Samples correspond to 4 μg total protein per lane each. (c) Exemplary negative‐staining electron microscopy of input material and fraction F1‐AP. Scale bars 200 nm. (d) EV concentration determined by NanoSight measurement. Data represent particles per mL of starting plasma determined in three independent experiments

**FIGURE 5:**
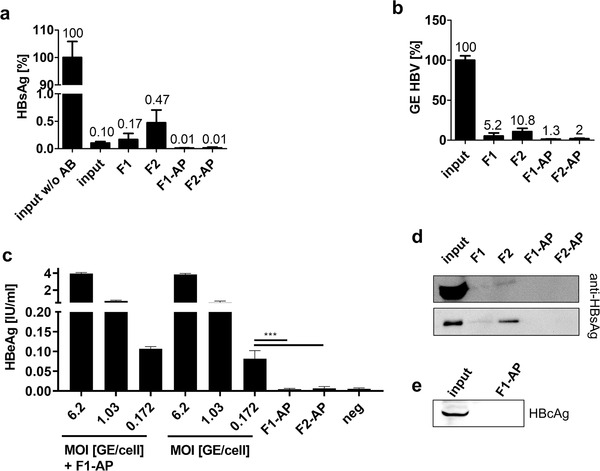
EVs purified by both size‐exclusion chromatography and affinity‐based purification are devoid of Hepatitis B Virus components. Plasma samples containing 3.1 × 10^8^ GE HBV and 767 μg anti‐HBsAg per ml plasma were subjected to SEC and affinity‐based purification. (a) HBsAg determined in input (plasma, HBV + anti‐HBsAg) and purified plasma samples is given as a percentage of input concentration. Samples correspond to 60 μl starting plasma each. Data represent three independent experiments in duplicates. (b) GE HBV determined in input and purified samples by qRT‐PCR is given as a percentage of input concentration. Samples correspond to 2.9 μl starting plasma each. Data represent three independent experiments in technical triplicates. (c) HepG2‐NTCP cells were treated with HBV in decreasing MOI, starting with an MOI of 6.2 vp/cell in six‐fold dilutions, and F1‐AP (MOI 0.174 vp/cell) and F2‐AP (MOI 0.266 vp/cell). HBeAg levels were measured by ELISA on day 5 post infection. Graph combines three independent experiments in duplicates. ^***^ *P* < 0.001 (d) Western blot analysis of residual anti‐HBsAg contaminations. Samples correspond to 6 μl starting plasma each (upper gel and individual samples F1, F2, F1‐AP and F2‐AP lower gel) or 0.02 μl starting plasma (input sample lower gel). One representative Western Blot out of three independent experiments is shown. (e) Western blot analysis of HBcAg. Samples were loaded at volumes corresponding 260 μl starting plasma each. One representative Western blot out of three independent experiments is shown

Since overloading the affinity column with antibodies lead to poorer purification results, it was essential to remove excess antibody that was not bound to HBV particles prior to affinity‐based purification. The antibody removal was achieved by applying SEC after antibody incubation and before affinity‐based purification. Before SEC, 300 μl input sample contained 180 μl plasma samples spiked with 5.56 × 10^7^ GE HBV and 138 μg anti‐HBsAg (equals 3.1 × 10^8^ GE HBV and 767 μg anti‐HBsAg per ml starting plasma), which was within the binding capacity of the affinity column (compare Figure 3). Removal of unbound antibody resulted in an actual antibody content far lower than the original 138 μg and guaranteed an optimal binding capacity (compare Figure 5d). The combination of SEC and affinity‐based purification resulted in pure EV samples (F1‐AP and F2‐AP) devoid of smaller contaminants as shown in size and electron microscopy (Figure 4a and 4c and Figure S4). Protein staining also showed enriched EV proteins, but no cellular proteins excluded from EVs (Figure 4b) (Thery et al., 2018). Particle numbers decreased by a 4.6‐fold (F1 to F1‐AP) and a 9.4‐fold (F2 to F2‐AP) after affinity‐based purification (Figure 4e). Consequently, the combination of SEC and affinity‐based purifications produced pure EV samples without detectable contaminants.

While EV purification was successful, it was not clear whether the purified EV samples retained functional HBV virions. HBsAg and HBcAg were not detectable anymore after combined SEC and affinity‐based purification as shown by ELISA and Western blot analysis (Figure 5a and e). The number of HBV genomes was profoundly decreased between the input sample and the purified EVs according to qPCR (Figure 5b). To determine the infectious potential of purified samples, HBV‐susceptible HepG2‐NTCP cells were treated with affinity‐purified EV‐fractions (Ko et al., 2018). Inoculum of F1‐AP equalled a MOI of 0.174 viral particles (vp)/cell and inoculum of F2‐AP equalled an MOI of 0.266 vp/cell according to GE detected in qRT‐PCR. No residual viral infectivity was observed after the combined SEC and affinity‐purification (F1‐AP and F2‐AP) (Figure 5c), though the applied MOI was located in the linear range of HBeAg‐release as shown by titration experiments (Figure S5). Cells were infected with either HBV or HBV mixed with affinity‐purified fraction 1 (HBV + F1‐AP) as a positive control, as residual anti‐HBsAg would interfere with downstream applications and viral infectivity. As HBV + F1‐AP samples showed no decreased infectivity compared to HBV samples, viral infection did not appear to be inhibited by residual unbound anti‐HBsAg. Further analysis revealed small amounts of anti‐HBsAg in SEC purified samples, which were most likely bound to HBV components. However, no anti‐HBsAg was found after combined SEC and affinity‐purification (Figure 5d).

Taken together, these results demonstrate that a combination of SEC and affinity‐based purification was effective at removing viral components and viral infectivity from EV samples. The resulting samples were also devoid of contaminating antibodies, and thus were suitable for downstream applications.

## 4 DISCUSSION

Virological research in the field of EVs has been limited by a lack of an adequate method to remove viruses from EV samples. Common EV preparation methods like SEC and density gradient centrifugation alone do not lead to sufficient viral removal from EV samples, as EVs overlap with virions because of similar size, density and surface composition (McNamara & Dittmer, 2019). Additionally, current affinity‐based purification protocols do not render pure EV samples that are useful for downstream application due to antibody contaminations. As a compromise, studies using recombinant virus strains, which did not express viral surface proteins and did not induce viral morphogenesis, ensured purity of EV samples but did not reflect natural infection conditions (Dreux et al., 2012; Jung et al., 2020). Our approach combined both SEC and affinity‐based separation specifically targeting viral surface antigens to facilitate the purification of EV samples devoid of virus or antibody contaminations.

To determine the potential of SEC for HBV removal, existing protocols were optimized and applied to the purification of virus‐containing plasma samples. HBV‐sized particles with a diameter of about 42 nm (Liu, 2014) were profoundly decreased in relation to EV‐sized particles. HBsAg concentrations were lowered but, most likely due to the inability of SEC to remove larger filaments, were not reduced as efficiently. SEC‐fraction F2, which contained smaller particles, consistently featured more HBV‐derived impurities compared to the earlier SEC‐fraction F1. Comparing the size distribution of the different samples F2 shown in Figures 2a and 4a, we observe a strong difference in the efficiency of particle removal < =  50.75 nm. We suspect that quality differences between different qEV lots are responsible for this effect and that our data show the full range of expected results. Depending on the experimental setup, it may be more reasonable to use F1 only, as it was not as affected by quality discrepancies. Nevertheless, HBV contamination could be completely eliminated by subsequent affinity purification, which underlines the robustness of affinity purification. Accordingly, we concluded that SEC clearly decreased the number of smaller virions in EV samples but that additional purification steps were required.

We next analysed the robustness of protein A‐based affinity purification in the removal of HBsAg‐containing particles. However, we considered non‐purified plasma as an ineligible input material for affinity purification due to its heterogeneous composition including diverse antibodies and virus‐sized very‐low density lipoprotein particles (Karimi et al., 2018; Simonsen, 2017). Visualization of virus removal by size measurement was ensured through the use of pure EV samples containing HBV and anti‐HBsAg as a starting material. We observed that affinity‐purification indeed removed HBV, HBsAg, HBV DNA genomes and anti‐HBsAg from EV samples, but saturation of antibody‐binding columns lead to the elution of both unbound antibody and antibody‐masked HBsAg. Using HBV‐spiked plasma samples instead of pre‐purified samples, protein A‐column capacity was clearly reduced. We suspect that the binding of antibodies other than anti‐HBsAg by protein A agarose beads is responsible for this effect. Protein A binds Fc‐parts of antibodies independently of the epitopes recognized by the variable parts. Non‐HBV binding antibodies would block binding sites on protein A agarose beads, thereby decreasing their HBV‐removing capacity. Consequently, the antibody input should be as low as possible while still ensuring the complete coating of HBV‐derived particles.

Combining these results, the addition of anti‐HBsAg to virus‐containing plasma prior to SEC should lead to the removal of excess antibody, thereby preventing the undesirable blocking of antibody binding sites and column overload. Furthermore, the low number of purification steps in this protocol also prevent an unnecessary loss of EVs. The combination of SEC and affinity‐purification results in pure EV samples that are devoid of virus‐sized particles, HBsAg, HBcAg, anti‐HBsAg or infectious virions.

The number of viral GE after purification was reduced below 2% of the input concentration. The residual GE detected by qPCR might correlate with the release of non‐HBsAg enveloped viral capsids by HBV producing cells (Arzberger et al., 2010). Likewise, viral genomes, capsids, and full virions enveloped by EV membranes instead of viral surface proteins could still be contained in the EV sample (Nolte‐’t Hoen et al., 2016), as the presence of HBV genomes in EVs after CD63‐dependent affinity‐based purification has already been reported (Yang et al., 2017). HBV immune escape‐mutants unbound by anti‐HBsAg cannot be removed in the described manner. It should be acknowledged that HBV could be removed from EV samples, but not vice versa.

The decrease in particle numbers after affinity‐based purification not only reflects the removal of virions, which are already greatly reduced by SEC, but also correlates to the removal of subviral particles, which outnumber HBV‐virions by a thousand‐fold, and are not completely removed by SEC due to heterogenous sizes (Ganem & Prince, 2004). Strikingly, F2 containing more virus‐sized particles, more HBsAg, and more anti‐HBsAg compared to F1 showed a greater decrease in particle numbers after affinity‐based purification. Of note, HBsAg content decreases stronger than particle yield (F1 to F1‐AP: 17 fold HBsAg loss vs. 4.6 fold particle loss; F2 to F2‐AP: 47 fold HBsAg loss vs. 9.4 fold particle loss). Consequently, removal of viral particles cannot be explained by loss of particle numbers in general.

One disadvantage of this method is that some EV populations will be lost or strongly reduced. For example, virus‐like particles carrying HBsAg on their surface will be removed from the EV sample. Therefore, the validity of this method is limited to particles whose infectivity is independent of viral surface protein. However, we expect the majority of EVs released from the infected cell not to contain HBsAg. The majority of HBsAg is incorporated into subviral particles that are formed at the ER membrane, not at the multivesicular bodies, and outnumber virions by more than a thousand‐fold (Patient et al., 2007; Tong & Revill, 2016). The secretion of subviral particles occurs via the Golgi network and the constitutive secretion pathway (Zeyen & Prange, 2018). Only a minority of HBV envelopes are transported to multivesicular bodies via uncharacterized pathways for complete virion formation. It is not reported that the plasma membrane is involved in the formation of the HBV envelope, so the formation of microvesicles should remain unaffected. It should also be mentioned that virus‐sized EVs will be greatly reduced, although not completely eliminated (Figure 2a, Figure 4a and Figure S6).

Our approach enables the complete removal of viral particles from EV samples under natural conditions for the first time. Regarding the robustness of this method, we used an HBV level of 3.1 × 10^8^ GE HBV/ml plasma in this experimental setup. In large‐scale clinical studies, about 80% of chronically HBV infected patients showed HBV DNA serum levels below 10^6^ GE HBV/ml serum and 90% showed serum levels below 10^8^ GE HBV/ml serum (Chen et al., 2006, 2009). Moreover, the described protocol does not seem to be at its limit, especially since the majority of HBV‐GE is removed by SEC, regardless of antibody binding capacities. Consequently, we assume that even larger amounts of HBV can be removed by our approach.

Unnecessary loss of EVs is minimized by nested purification steps. A time‐saving and reproducible workflow, as well as the absence of interfering antibodies, should permit application of this methodology for future functional studies. By using appropriate antibodies, the basic principle of this procedure could also potentially be transferable to viruses other than HBV. Virus‐free EVs could also potentially be purified from the sera of infected patients, enabling the study of the impact of EVs on diagnostics for viral infections. These results facilitate further studies, which can examine the role of EVs in viral infections with a particular regard to viral spread and EV‐mediated immune responses to viral infections.

To summarize, we developed a comprehensive approach for the complete removal of HBV from EV samples. The resulting EV samples were utterly devoid of virus‐sized particles, viral surface antigen, viral core antigen, antibody contaminations and infectious potential. Although the yield after combined purification steps is low, we estimate that the protocol can be upscaled to obtain enough EVs for functional studies. In previous studies, we successfully used SEC‐purified EVs from 90 μl serum samples of hepatitis D virus (HDV) infected patients per 96 well format based immune stimulation in duplicates (Jung et al., 2020). As additional affinity purification decreases particle numbers in EV sample F1 by about a five‐fold, 500 μl plasma sample should render enough EVs for one immune stimulation in duplicates. Likewise, we already performed immune stimulations using EVs released by HDV monoinfected cells, eliciting a proinflammatory cytokine response in primary human immune cells (Jung et al., 2020). With EVs obtained within 2 weeks from infected cells in two 175 cm^2^ cell culture flasks, five immune stimulations could be performed in concentration gradients and duplicates. In sum, the protocol presented here should enable functional studies on the virological impact of EVs under natural conditions without confounding virions or antibody‐mediated effects. Further studies on EV‐mediated effects in viral infections could lead to the development of new diagnostic procedures or new therapies against various diseases.

### 4.1 Geolocation information

Institute for Virology, Helmholtz Zentrum München/Technical University of Munich, School of Medicine, Trogerstraße 30, D‐81675 München, Germany, 48°08′19.0“N 11°36′06.0″E.

## CONFLICTS OF INTEREST

The position of Daniela Stadler was in part financed by a research grant from ALIOS BioPharma.

## Supporting information

Supporting Information

Supporting Information

Supporting Information

Supporting Information

Supporting Information

Supporting Information

## Data Availability

Data sharing not applicable to this article as no datasets were generated or analysed during the current study.
