# Supplementary figures and images for: Efficient and reproducible depletion of hepatitis B virus from plasma derived extracellular vesicles
Source: J Extracell Vesicles. 2020 Dec 22;10(2):e12040. doi: 10.1002/jev2.12040 (PMC7754750; doi:10.1002/jev2.12040)

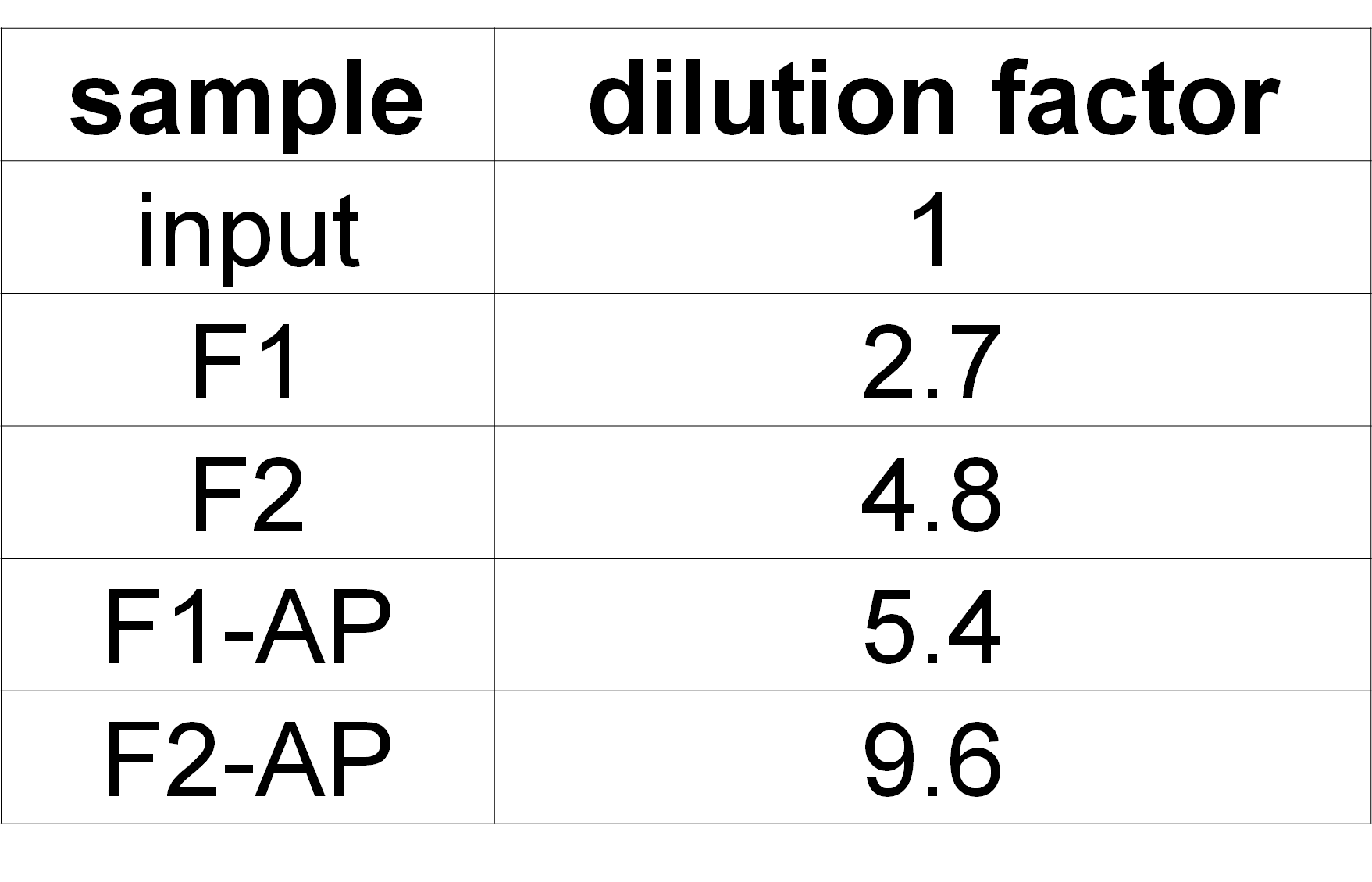

Supplement: Supplementary file 1 — Supporting Information [file JEV2-10-e12040-s001.tif]

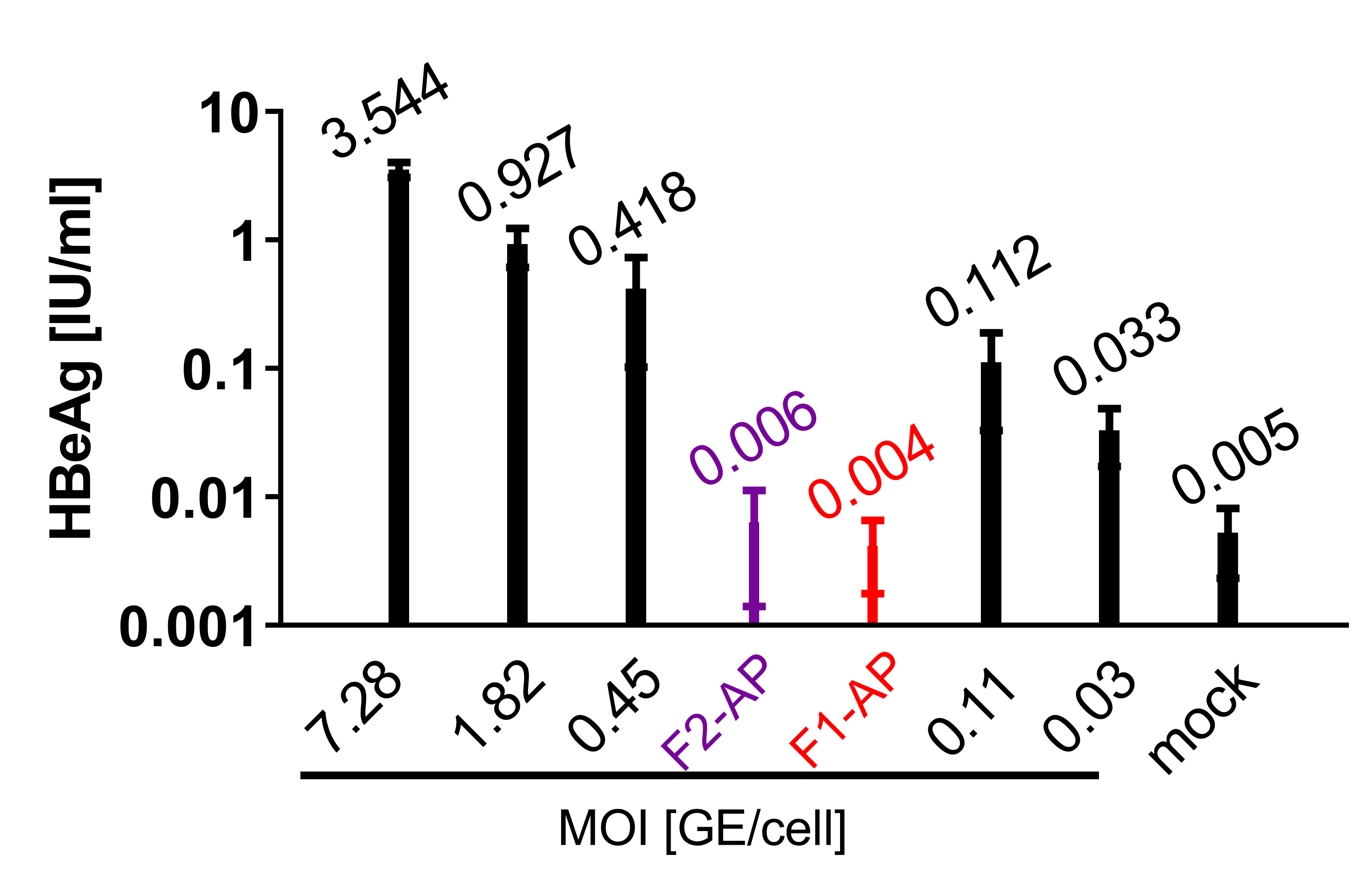

Supplement: Supplementary file 5 — Supporting Information [file JEV2-10-e12040-s005.tif]
